# Akutes Abdomen – seltene Ursache bei einer 80-jährigen Patientin unter immunsuppressiver Therapie

**DOI:** 10.1007/s00108-023-01593-z

**Published:** 2023-10-13

**Authors:** J. Schlottmann, S. Miller, C. Scheurig-Münkler, C. Merkl, T. Weber, S. Eser, A. Fuchs, H. Messmann, A. Probst

**Affiliations:** 1 3. Medizinische Klinik, Universitätsklinikum Augsburg, Stenglinstraße 2, 86156 Augsburg, Deutschland; 2 Institut für Pathologie und molekulare Diagnostik, Universitätsklinikum Augsburg, Augsburg, Deutschland; 3 Klinik für Diagnostische und Interventionelle Radiologie und Neuroradiologie, Universitätsklinikum Augsburg, Augsburg, Deutschland; 4 Klinik für Allgemein‑, Viszeral- und Transplantationschirurgie, Universitätsklinikum Augsburg, Augsburg, Deutschland

**Keywords:** Nocardiose, Autoimmunhepatitis, Abdominelle Nocardiose, Abszess, „Omental cake“, Nocardiosis, Autoimmune hepatitis, Abdominal nocardiosis, Abscess, Omental cake

## Abstract

Eine 80-jährige Frau stellte sich zur Abklärung abdomineller Schmerzen vor. Vorausgegangen war die Diagnosestellung einer Autoimmunhepatitis mit Einleitung einer immunsuppressiven Therapie und Auftritt zweier Pneumonien mit opportunistischen Erregern. Die Bildgebung erbrachte einen „omental cake“ mit Verdacht auf Peritonealkarzinose. Bei Auftritt eines akuten Abdomens erfolgte eine explorative Laparotomie, hierbei zeigten sich intraabdominelle Abszesse. Anhand von Blutkulturen und des intraoperativ gewonnenen Materials wurde eine disseminierte Nocardiose diagnostiziert. Die Patientin verstarb aufgrund einer fulminant verlaufenen Sepsis.

## Abstract (en)

An 80-year-old woman presented to the emergency department due to abdominal pain. She had a history of opportunistic pneumonia under the effects of immunosuppression after the diagnosis of autoimmune hepatitis. The imaging showed an omental cake formation and the suspicion of peritoneal carcinomatosis. The patient developed an acute abdomen during the hospital stay, followed by exploratory laparotomy. In the presence of extensive intra-abdominal abscess formation both surgically acquired material and blood culture revealed disseminated nocardiosis. The course was fatal due to fulminant septic shock.

## Anamnese

Bei einer 80-jährigen Patientin wurde im Juli 2021 eine Autoimmunhepatitis anhand typischer Laborbefunde (ANA-IIFT 1:400; ANA-Screening 2. Muster 1:3200; Sp100-AK positiv, p‑ANCA-IIFT 1:1024) und Histologie (chronisch aktive, überwiegend portale Hepatitis mit Übergreifen auf die Intermediärzone sowie periportale Fibrose) diagnostiziert und mittels Prednisolon (initial 40 mg/Tag) und Azathioprin (50 mg/Tag – entsprechend 1,5 mg/kg KG) therapiert. Nach Ansprechen folgten im Verlauf erneute Hospitalisierungen bei Herpes-simplex(HSV)-1-Pneumonie und pulmonaler Aspergillose (HR-CT Thorax mit kleinflächigem Infiltrat am kleinen Lappenspalt rechts mit Milchglastrübung; bronchoalveoläre Lavage [BAL] mit Nachweis von 248.000 HSV-1-Viruskopien/ml, später BAL-Nachweis von *Aspergillus*-Antigen und Kultur von *Aspergillus fumigatus*). Diese wurden mittels Aciclovir (1200 mg/Tag über 14 Tage) und Voriconazol (400 mg/Tag für zunächst 6 Wochen) therapiert. Aufgrund der immunsuppressionsassoziierten Infektionen erfolgte eine Immundiagnostik mit Nachweis einer absoluten CD4^+^-T-Lymphozytenzahl von 96 Zellen/µl. Die Kombinationstherapie wurde beendet und eine Erhaltungstherapie mittels Budesonid eingeleitet (3 × 3 mg/Tag). Sonstige Vorerkrankungen lagen nicht vor.

## Klinischer Befund

09/21 erfolgte die erneute Vorstellung der Patientin, seit dem Vorabend waren Bauchschmerzen und Fieber aufgetreten. Das Abdomen zeigte sich über allen Quadranten druckschmerzhaft. Die Körpertemperatur betrug 38,3 °C tympanal. Die Therapie bestand aus Budesonid 9 mg/Tag und Voriconazol 400 mg/Tag.

## Labordiagnostik (Tab. 1)

**Table 1:** 

P Natrium (ISE)*	136–145	mmol/l	*134*↓
P Kalium (ISE)	3,5–5,1	mmol/l	*3,31*↓
P Kalzium	2,2–2,55	mmol/l	*1,99*↓
Geschätzte GFR (CKD-EPI)**	> 90	ml/min pro 1,73 m^2^	*64,0*↓
P Bilirubin direkt	0–0,3	mg/dl	^*a*^*0,39*↑
P CRP	0–0,5	mg/dl	*21,41*↑
Leukozyten	3,0–10,0	/nl	**13,44**↑
Erythrozyten	4,1–5,3	/pl	**3,01**↓
Hämoglobin	120–160	g/l	**108**↓

*Ionenselektive Elektrode

**Glomeruläre Filtrationsrate nach „Chronic Kidney Disease Epidemiology Collaboration“ Formel

Pfeilmarkierungen entsprechend Abweichung von Referenzwerten

## Weitere Diagnostik

**Figure 1:**
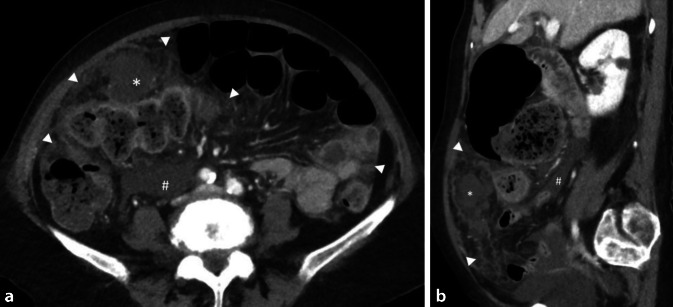

Im Röntgen des Thorax zeigten sich Infiltrate im rechten Mittelfeld und beiden Unterfeldern. Aufgrund des ausgeprägten klinischen Befunds folgte eine CT des Abdomens mit Verdacht auf Peritonealkarzinose und wenig Aszites. Es zeigte sich das Bild eines Retentionsmagens bei vermutetem CUP(„cancer of unkown primary“)-Syndrom, DD infektiös-entzündliches Geschehen. Weiter fanden sich Wandverdickungen und -betonung des gesamten Kolonrahmens und der Verdacht auf Dünndarmsubileus (Abb. 1).

## Weiteres Vorgehen

Seitens Chirurgie wurde aufgrund des Malignitätsverdachts zunächst keine Indikation zur operativen Therapie gestellt. Unter stuhlregulierenden Maßnahmen kam es zunächst zu einer Beschwerdebesserung. Eine kalkulierte antibiotische Therapie mittels Piperacillin/Tazobactam wurde nach Asservierung von Blutkulturen eingeleitet. Es erfolgte die stationäre Aufnahme in die Gastroenterologie. Am Folgetag zeigte sich eine diffuse Abwehrspannung mit folgender Labordiagnostik (Tab. 2):

**Table 2:** 

P Harnstoff	< 71	mg/dl	*94*↑
P Kreatinin (Jaffé)	0,5–0,9	mg/dl	*2,07*↑
Geschätzte GFR (MDRD)	> 60	ml/min pro 1,73 m^2^	*24,5*↓
Geschätzte GFR (CKD-EPI)	> 90	ml/min pro 1,73 m^2^	*22,1*↓
P Cholinesterase	5320–12.920	U/l	*992*↓
P CRP	0–0,5	mg/dl	*32,83*↑
P Prokalzitonin	0–0,5	ng/ml	^a^*28*↑↑
P Albumin	35–52	g**/1**	*15,7*↓
P Osmolalität	280–300	mosmol/kg	*302*↑
Erythrozyten	4,1–5,3	/pl	**3,03**↓
Hämoglobin	120–160	g/l	**108**↓
Hämatokrit	36–45	%	**31,5**↓
MCV	82–101	fl	**104,0**↑
MCH	27–34	pg	**35,6**↑
Thrombozyten	140–440	/nl	**139**↓

In der erneuten chirurgischen Konsultation wurde aufgrund des progredienten Erkrankungsbilds die Indikation zur Notfalllaparotomie gestellt, die noch am selben Tag erfolgte.

Der Befund wurde als Abszess im Omentum majus des rechten Oberbauchs mit Peritonitis und kleinknotigen Raumforderungen am gesamten Dünndarmmesenterium und paralytischem Ileus zusammengefasst.

Während des postoperativen Verlaufs auf der Intensivstation kam es zu einem fulminanten Schockgeschehen und Versterben der Patientin.

## Mikrobiologie

**Figure 2:**
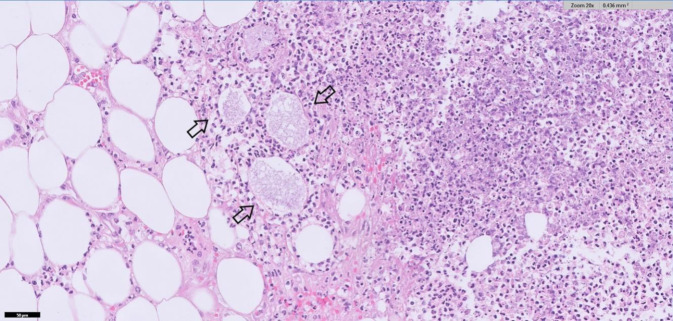

Die Blutkulturen und Op.-Materialien (Abstrich aus Bauchhöhle und Omentum) erbrachten den Nachweis von *Nocardia farcinica*. In der Obduktion fand sich eine Peritonitis mit Herden im gesamten Bauchraum, auf Organen, Fettgewebe und Verwachsungen des Dünn- und Dickdarms (Abb. 2 und 3). Histologisch handelte es sich um Gram- und Grocott-positive Erreger. Pulmonal zeigten sich eine akute Bronchopneumonie sowie multiple granulomartige Abszesse (Abb. 4). Im Urogenitalsystem fanden sich Abszesse in der Niere und im perirenalen Fettgewebe. In der Leber zeigten sich eine Fibrose und beginnende kleinknotige Leberzirrhose. Als Todesursache wurde ein infektiös-toxisches Herz-Kreislauf-Versagen bei Sepsis aufgrund disseminierter Nocardiose mit septikopyämischen Herden in Thorax, Abdomen und Retroperitoneum konstatiert.

**Figure 3:**
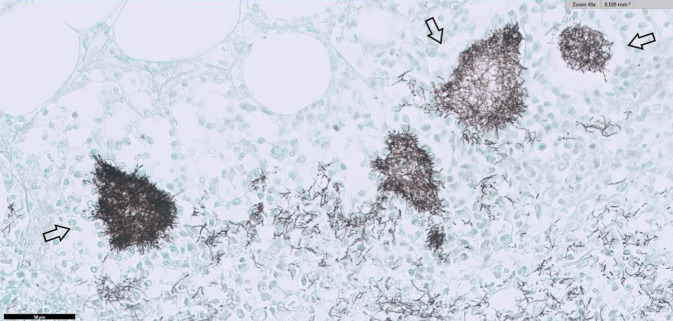

**Figure 4:**
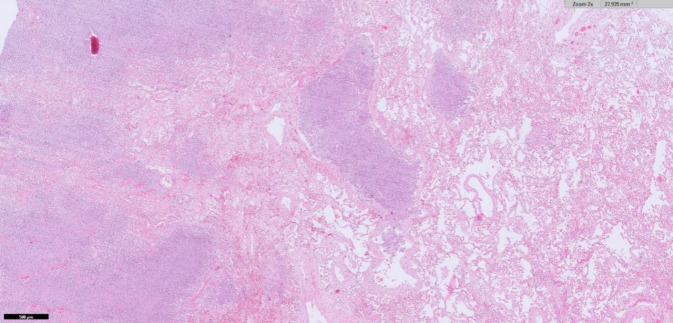

## Diskussion

Nocardien sind obligat aerobe, stäbchenförmige, grampositive Bakterien. Die Gattung *Nocardia* hat über 50 Spezies, eine zunehmende Zahl wird als potenziell pathogen für den Menschen eingestuft [2, 7]. Nocardien kommen in stehenden Gewässern, Tierfäkalien und im Boden ubiquitär vor [2]. Die Infektion erfolgt durch Inhalation oder Wundkontamination.

Risikofaktoren für die Entwicklung von Nocardiosen sind onkologische Leiden, Immunsuppression sowie zugrunde liegende Lungenerkrankungen. In 20–30 % der Fälle kann keine Immundefizienz nachgewiesen werden [2, 7, 9].

Der kulturelle Nachweis dauert etwa 2 Wochen, der Nachweis aus Blutkulturen gelingt selten – eine Alternative bietet die Polymerase-Kettenreaktion [2, 3, 5, 7].

Pulmonale und disseminierte Nocardiosen verlaufen schwerwiegend, bei Immunsuppression verlaufen trotz adäquater Therapie über 50 % der disseminierten Nocardiosen letal [9].

Als Therapie der Wahl wird Trimethoprim/Sulfamethoxazol (TMP/SMX) mit 15 mg/kg pro Tag oder eine hoch dosierte Sulfonamidmonotherapie (z. B. Sulfadiazin 1 g/4–6 h) beschrieben. Bei Immunsuppression und disseminierter Erkrankung sollte TMP/SMX zusammen mit Amikacin, Imipenem oder Meropenem verabreicht werden, bis ein Antibiogramm vorliegt – für viele Spezies sind Resistenzinterpretationen möglich [1]. Die Empfehlung zur Dauer der Therapie variiert (nach Ausprägung der Erkrankung und Immunstatus) zwischen 3 und 12 Monaten. Ab einer Therapiedauer von 3 Monaten sind Rezidive selten, unter Immunsuppression soll grundsätzlich 12 Monate behandelt werden [7, 10].

Eine Drainageanlage bei Abszessen sowie antimikrobielle Therapie ist häufig ausreichend, selten bedarf es operativer Sanierung [6].

Als häufigste Präsentationen sind Wundinfektionen, pulmonale Infektionen oder zerebrale Abszedierungen beschrieben. Isolierte abdominelle/retroperitoneale Nocardiosen sind äußerst selten mit weltweit wenigen Fällen [4, 9]. Infektionen bei peritonealer Dialyse wurden in seltenen Fällen beschrieben [8, 9]. Etwas häufiger finden sich disseminierte Nocardiosen mit abdomineller Beteiligung nach Transplantation oder Immunsuppression [1, 8].

Die häufigsten nachgewiesenen Spezies bei disseminierten Nocardiosen mit abdomineller Beteiligung waren *N. asteroides* und *N. farcinica*, wie auch in dem Fall [9].

Die Diagnosestellung ist oft schwierig; häufig wird die abdominelle Nocardiose als Peritonealkarzinose, abdomineller Tumor oder Tuberkulose interpretiert [1, 7]. Hier wurde eine Peritonealkarzinose vermutet, die anhand des Akutverlaufs und der diesbezüglich unauffälligen Sonographiebefunde in der Vorgeschichte unwahrscheinlich erschien. Beachtenswert ist die geringe Dosierung der Immunsuppression, unter der Infektion und fulminanter Verlauf auftraten. Seit 2000 sind ähnliche Verläufe unter intensiver Immunsuppression, nicht jedoch unter oraler Budesonidtherapie berichtet [9].

Die initiale Therapie der autoimmunen Lebererkrankung erfolgte leitliniengerecht mit Prednisolon und Azathioprin. Diskussionswert ist, ob Azathioprin zum Zeitpunkt der Infektion noch residual wirksam war und zum Verlauf beigetragen hat; zwischen Absetzen und Nachweis der Nocardiose lagen 18 Tage. Spekulativ bleibt, ob ein früheres Absetzen der Kombinationstherapie oder eine alleinige Budesonidtherapie den Verlauf hätte verhindern können.

## Fazit für die Praxis

Die Nocardiose ist eine seltene Infektionserkrankung. Wundinfekte, pulmonale Infektionen sowie zerebrale Abszedierung sind häufiger, allerdings finden sich bei disseminierter Nocardiose seltener auch peritoneale und retroperitoneale Beteiligungen. Betroffen sind überwiegend Patienten unter Immunsuppression, mit Lungenerkrankungen oder peritonealer Dialyse. Bereits niedrige Dosen immunsuppressiver Therapie sind ein Risiko für schwerwiegende Verläufe. Die Diagnosestellung basiert auf der Identifikation des Erregers in gewonnenem Material.

## References

[1] Abreu C, Rocha-Pereira N, Sarmento A, Magro F (2015). Nocardia infections among immunomodulated inflammatory bowel disease patients: a review. World J Gastroenterol.

[2] Brown-Elliott BA, Brown JM, Conville PS, Wallace RJ (2006). Clinical and laboratory features of the nocardia spp. based on current molecular taxonomy. Clin Microbiol Rev.

[3] Couble A, Rodríguez-Nava V, de Montclos MP, Boiron P, Laurent F (2005). Direct detection of nocardia spp. in clinical samples by a rapid molecular method. J Clin Microbiol.

[4] Hammoud M, Kraft C, Pulst-Korenberg J, Chenoweth C, Gregg KS (2014). Disseminated nocardia paucivorans infection in an immunocompetent host. Infection.

[5] Kontoyiannis DP, Ruoff K, Hooper DC (1998). Nocardia bacteremia. Report of 4 cases and review of the literature. Medicine.

[6] Lee GY, Daniel RT, Brophy BP, Reilly PL (2002). Surgical treatment of nocardial brain abscesses. Neurosurgery.

[7] Lerner PI (1996). Nocardiosis. Clin Infect Dis.

[8] Matchett C, Djamali A, Mandelbrot D, Saddler C, Parajuli S (2019). Nocardia infection in kidney transplant recipients: a single-center experience. Transpl Infect Dis.

[9] Tramèr L, Mertz KD, Huegli R, Hinic V, Jost L, Burkhalter F, Wirz S, Tarr PE (2020). Intra-abdominal nocardiosis-case report and review of the literature. J Clin Med.

[10] Wallace RJ, Septimus EJ, Williams TW, Conklin RH, Satterwhite TK, Bushby MB, Hollowell DC (1982). Use of trimethoprim-sulfamethoxazole for treatment of infections due to nocardia. Rev Infect Dis.

